# Classification of Insanity

**Published:** 1887-03

**Authors:** 


### CLASSIFICATION OF INSANITY.

Professor Lefebvre's classification, approved by the Belgian Society:

1. Idiocy.
2. Cretinism.
3. Dementia Paralytica.
4. Dementia.
5. Toxical Alienation.
6. Mania.
7. Melancholia.
8. Folie Circulaire.

The German plan, quoted by Meynert, as hinted at by Guttstadt, and substantially adopted at the conference of German alienists, at Frankfort-on-the-Main, in 1881:

1. Ordinary Mental Disorder.
2. Paralytical Mental Disorder.
3. Mental Disease, accompanied by Epilepsy.
4. Imbecility, Idiocy and Cretinism.
5. Alcoholic Mania.
6. Individuals who need Watching.

Westphal's plan, cited by Meynert, and approved by Weisbaden conference of 1873:

1. Melancholia.
2. Mania.
3. Secondary Mental Disease.
4. Paralytical Mania.
5. Epileptic Mania.
6. Imbecility, Idiocy and Cretinism.
7. Delirium Tremens (Toxie).

### MEYNERT'S PLAN OF CLASSIFICATION.

As submitted to the Austria-Hungarian Conference.

Simple Mental Disorder:

Acute. { Melancholia. / Mania. / Insanity. / Primary Imbecility.

| | |
|---|---|
| Chronic. | Primary Insanity.<br>Intermittent Mental Disease.<br>Secondary Mental Disease. |
| Complicated Mental Diseases. | Paralytical.<br>Epileptical.<br>Hystero-Epileptic.<br>Mania. |
| Toxic. | Alcoholic.<br>Other Toxic Agents. |
| Individuals who need watching. | Attempts at Suicide.<br>Crimes, etc., etc. |

Dr. D Hack Tuke, of England, had submitted a classification of mental diseases, which had been unanimously adopted by the Administrative Council of the British Medico-Pyschological Association, which is as follows:

1. Mental Alienation.
   Congenital or acquired.
   Idiocy, Imbecility and Cretinism.
   With Epilepsy—without Epilepsy.
2. Epilepsy.
3. General Paresis.

| | |
|---|---|
| 4. Mania. | Acute.<br>Chronic.<br>Recurrent or periodic.<br>A potu—.<br>Puerperal.<br>Senile. |
| 5. Melancholia. | Acute.<br>Chronic.<br>Recurrent.<br>Puerperal.<br>Senile. |
| 6. Dementia. | Primary.<br>Secondary.<br>Senile.<br>Organic (tumors, hemorrhage). |

7. Chronic Delirium (Monomania).

8. Moral Insanity.

Dr. D. Hack Tuke stated that recurrent insanity had not been included, because it required usually one year of observation before it could be certainly diagnosed, and an International classification should be as simple as possible, giving freedom to each country to add supplementary divisions if desired.—*Alienist and Neur.*

